# Chorioamnionitis induces enteric nervous system injury: effects of timing and inflammation in the ovine fetus

**DOI:** 10.1186/s10020-020-00206-x

**Published:** 2020-09-03

**Authors:** C. Heymans, I. H. de Lange, K. Lenaerts, L. C. G. A. Kessels, M. Hadfoune, G. Rademakers, V. Melotte, W. Boesmans, B. W. Kramer, A. H. Jobe, M. Saito, M. W. Kemp, W. G. van Gemert, T. G. A. M. Wolfs

**Affiliations:** 1 Department of Surgery, NUTRIM School of Nutrition and Translational Research in Metabolism, Maastricht University, Maastricht, the Netherlands; 2 Department of Pediatrics, School for Oncology and Developmental Biology (GROW), Maastricht University, P.O. Box 616, Universiteitssingel 50, 6200 MD Maastricht, The Netherlands; 3 Department of Pathology, School for Oncology and Developmental Biology (GROW), Maastricht University Medical Center, Maastricht, the Netherlands; 4 Biomedical Research Institute, Hasselt University, Hasselt, Belgium; 5 Neonatology, Department of Pediatrics, Maastricht University Medical Center, Maastricht, the Netherlands; 6 Division of Obstetrics and Gynecology, The University of Western Australia, Crawley, Australia; 7 Division of Neonatology/Pulmonary Biology, The Perinatal Institute, Cincinnati Children’s Hospital Medical Center, University of Cincinnati, Cincinnati, OH USA; 8 Center for Perinatal and Neonatal Medicine, Tohoku University Hospital, Sendai, Japan; 9 School of Veterinary and Life Sciences, Murdoch University, Perth, Western Australia Australia; 10 Pediatric surgery, Department of Surgery, Maastricht University Medical Center, Maastricht, the Netherlands; 11 Department of Surgery, University Hospital Aachen, Aachen, Germany; 12 Department of Biomedical Engineering (BMT), Maastricht University, Maastricht, the Netherlands

**Keywords:** LPS, Intra-amniotic infection, Chorioamnionitis, Enteric nervous system, Sheep, Preterm birth, Necrotizing enterocolitis

## Abstract

**Background:**

Chorioamnionitis, inflammation of the chorion and amnion, which often results from intrauterine infection, is associated with premature birth and contributes to significant neonatal morbidity and mortality, including necrotizing enterocolitis (NEC). Recently, we have shown that chronic chorioamnionitis is associated with significant structural enteric nervous system (ENS) abnormalities that may predispose to later NEC development. Understanding time point specific effects of an intra-amniotic (IA) infection on the ENS is important for further understanding the pathophysiological processes and for finding a window for optimal therapeutic strategies for an individual patient. The aim of this study was therefore to gain insight in the longitudinal effects of intrauterine LPS exposure (ranging from 5 h to 15 days before premature delivery) on the intestinal mucosa, submucosa, and ENS in fetal lambs by use of a well-established translational ovine chorioamnionitis model.

**Methods:**

We used an ovine chorioamnionitis model to assess outcomes of the fetal ileal mucosa, submucosa and ENS following IA exposure to one dose of 10 mg LPS for 5, 12 or 24 h or 2, 4, 8 or 15 days.

**Results:**

Four days of IA LPS exposure causes a decreased PGP9.5- and S100β-positive surface area in the myenteric plexus along with submucosal and mucosal intestinal inflammation that coincided with systemic inflammation. These changes were preceded by a glial cell reaction with early systemic and local gut inflammation. ENS changes and inflammation recovered 15 days after the IA LPS exposure.

**Conclusions:**

The pattern of mucosal and submucosal inflammation, and ENS alterations in the fetus changed over time following IA LPS exposure. Although ENS damage seemed to recover after prolonged IA LPS exposure, additional postnatal inflammatory exposure, which a premature is likely to encounter, may further harm the ENS and influence functional outcome. In this context, 4 to 8 days of IA LPS exposure may form a period of increased ENS vulnerability and a potential window for optimal therapeutic strategies.

## Background

Chorioamnionitis, inflammation of the chorion and amnion during pregnancy, is associated with premature birth and contributes to significant neonatal morbidity and mortality (Galinsky et al. 2013; Goldenberg et al. 2000; Kim et al. 2015). Chorioamnionitis typically results from a bacterial infection ascending through the birth canal (Goldenberg et al. 2000). It is often clinically silent and therefore difficult to diagnose, but can nevertheless affect the developing fetus (Gantert et al. 2010). As the fetus swallows the amniotic fluid (AF), the intestine is directly exposed to bacterial components and inflammatory cytokines present in the AF, which can consequently cause gut injury and inflammation (Wolfs et al. 2014). Moreover, during chorioamnionitis, the fetus can develop a fetal inflammatory response syndrome (FIRS), which is characterized by increased systemic interleukin 6 (IL-6) and interleukin 8 (IL-8) levels (Gussenhoven et al. 2018). FIRS is an independent risk factor for considerable neonatal morbidity, including the postnatal intestinal disease necrotizing enterocolitis (NEC) (Gantert et al. 2010; Been et al. 2013). NEC has a high mortality of overall 25% with both significant short-term and long-term morbidity (Neu and Walker 2011). Severe intestinal inflammation is associated with NEC and can result in gut necrosis (Neu and Walker 2011; Neu and Pammi 2018). Gut specimens from NEC patients contain alterations in the enteric nervous system (ENS) including a loss of neurons and glial cells (Sigge et al. 1998; Wedel et al. 1998; Fagbemi et al. 2013; Zhou et al. 2013). The ENS resides in the intestinal wall and consists of two plexuses; the submucosal and myenteric plexus (Furness 2012). It operates autonomously and regulates diverse gastrointestinal functions such as motility, secretion, absorption and maintenance of gut integrity (Furness 2012). ENS development is a complex process that requires coordinated migration, proliferation and differentiation of the involved cell types, directed outgrowth of neurites and the establishment of an interconnected neuronal and glial cell network (Rao and Gershon 2018; Lake and Heuckeroth 2013). Importantly, ENS development continues in the early postnatal period (Hao et al. 2016; Burns et al. 2009) during which it is shaped by amongst others immune cells, microbiota and enteral nutrition (Hao et al. 2016).

Recently, we have shown in a preclinical ovine model that chronic chorioamnionitis is associated with significant structural ENS abnormalities (Heymans et al. 2020). Importantly, these alterations corresponded with those found in infants with NEC, indicating that ENS changes following chorioamnionitis may predispose to later NEC development (Heymans et al. 2020). Since inflammation is a dynamic process and the vulnerability of the fetus to injurious exposure during intra-uterine development varies, ENS alterations in response to inflammation can be time-dependent. As chorioamnionitis is often clinically silent and infants born after chorioamnionitis have been exposed to varying durations of intra-uterine inflammation, understanding time-dependent effects of intra-uterine inflammation on the ENS is clinically important to define optimal therapeutic strategies. Therefore, the aim of this study was to evaluate the time-dependent effects of 5 h to 15 days of intrauterine LPS exposure before premature delivery, on the intestinal submucosa, mucosa and ENS in fetal sheep.

## Methods

### Animal model and experimental procedures

The experiments were approved by the animal ethics/care committee of the University of Western Australia (Perth, Australia; ethical approval number: RA/3/100/928).

**Fig. 1:**
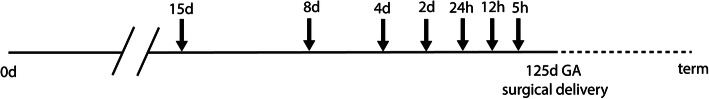
Study design. Pregnant ewes received an IA injection with 10 mg LPS at 5, 12, or 24 h or 2, 4, 8 or 15 days (black arrows) before preterm delivery at 122 days of gestation (term ~ 150 days). Control animals received an IA saline injection at comparable time points to LPS injections. Timing shown in gestational days

The ovine model and experimental procedures were previously described (Gussenhoven et al. 2018; Kuypers et al. 2013). In brief, 52 time-mated merino ewes carrying singleton fetuses were randomly assigned to eight different groups of six to seven animals. The pregnant ewes were IA injected under ultrasound guidance with 10 mg *Escherichia coli*-derived LPS (O55:B5; Sigma-Aldrich, St. Louis, MO, USA) dissolved in saline at 5, 12, or 24 h, or 2, 4, 8 or 15 days before preterm delivery at 125 days of gestation (equivalent of 30–32 weeks of human gestation for the gut; term gestation in sheep around 150 days). The study design is based on the clinically relevant situation that the gestational age of the infant is known, but not the length of exposure to inflammation. Hence, all samples were collected at the same gestational age and inflammation was induced at various times before sampling. Of importance, with a half-life time of 1.7 days, LPS persists in AF and can still be detected at 15 days (Newnham et al. 2003). A group receiving IA injections of saline at variable gestational ages comparable to LPS injections, ranging from 5 h to 15 days before preterm delivery, served as the controls (Fig. 1).

Fetuses were delivered by cesarean section at 125 days of gestation and immediately euthanized with intravenous pentobarbitone (100 mg/kg). Fetuses of both sexes were used. At necropsy, the terminal ileum was sampled and fixed in 10% formalin or snap frozen. Formalin-fixed tissues were subsequently embedded in paraffin.

### Antibodies

For immunohistochemistry, the following antibodies were used: polyclonal rabbit anti-myeloperoxidase ([MPO]; A0398, Dakocytomation, Glostrup, Denmark) for identification of neutrophils, polyclonal rabbit anti-bovine protein gene product 9.5 ([PGP9.5]; Z5116, Dakocytomation) for the detection of enteric neurons, polyclonal rabbit anti-doublecortin (Ab18723, Abcam, Cambridge, UK) for the detection of immature neurons, polyclonal rabbit anti-glial fibrillary acidic protein ([GFAP]; Zo334, Dakocytomation) for identification of activated enteric glial cells and polyclonal rabbit anti-S100β (PA5–16257, Invitrogen, Carlsbad, CA, USA) which is considered a general marker of enteric glial cells.

The following secondary antibodies were used: peroxidase-conjugated polyclonal goat anti-rabbit (111–035-045, Jackson, WestGrove, PA, USA) (MPO), peroxidase-conjugated polyclonal swine anti-rabbit (P0399, DakoCytomation) (doublecortin) and BrightVision+ Poly-HRP-Anti Mouse/Rabbit IgG Biotin-free (ImmunoLogic, Duiven, the Netherlands) (PGP9.5), and biotin conjugated polyclonal swine anti-rabbit (E0353, DakoCytomation) (GFAP, S100β).

### Immunohistochemistry

Paraffin embedded formalin-fixed terminal ileum was cut into 4 μm sections. Following deparaffinization and rehydration, sections were incubated in 0.3% H_2_O_2_ diluted in phosphorylated buffer saline ([PBS]; pH 7.4) to block endogenous peroxidase activity. For PGP9.5, doublecortin and S100β, antigen retrieval was achieved with citrate buffer. Non-specific binding was blocked for 30 min at room temperature with 10% normal goat serum (NGS) in PBS (MPO), 5% NGS in PBS (doublecortin), or 5% bovine serum albumin (BSA) in PBS (GFAP and S100β) or for 10 min at room temperature with 20% fetal calf serum (FCS) in PBS (PGP9.5). Subsequently, sections were incubated with the primary antibody of interest for 1 hour (MPO) or overnight (others) followed by the secondary antibody for 30 min (MPO) or 1 hour (others). MPO, PGP9.5 and doublecortin were recognized using a peroxidase-conjugated secondary antibody; antibodies against GFAP and S100β were detected with avidin-biotin complex (Vectastain Elite ABC kit, Bio-connect, Huissen, the Netherlands). Substrate staining was performed with 3-amino-9-ethylcarbazole ([AEC]; Merck, Darmstadt, Germany) (MPO), nickel-DAB (GFAP) or DAB (PGP9.5, doublecortin and S100β). Hematoxylin (MPO, PGP9.5, doublecortin and S100β) or nuclear fast red (GFAP) were used as nuclear counterstains.

### Quantification of immunohistochemical stainings

The Ventana iScan HT slide scanner (Ventana Medical Systems, Oro Valley, AZ, USA) was used to scan stained tissue sections. With the use of Pannoramic Viewer (version 1.15.4, 3DHISTECH, Budapest, Hungary), an overview picture of the transverse section of the ileum was taken. Two investigators blinded to the experimental groups counted the number of mucosal MPO-positive cells. Leica QWin Pro (version 3.4.0, Leica Microsystems, Mannheim, Germany) was used to calculate the mucosal surface area. The average number of mucosal MPO-positive cells corrected for total mucosal tissue surface area is reported as MPO-positive cells per area per animal. Secondly, random images of the submucosal layer were taken (200x). In five non-overlapping high power fields, the number of submucosal MPO-positive cells was counted by two investigators blinded to the experimental groups. The average number of submucosal MPO-positive cells per animal of the five power fields is reported as MPO-positive cells per area. For PGP9.5, doublecortin, GFAP and S100β, the surface of positively stained areas in the submucosal and myenteric ganglia and total surface area of the muscle layer were measured (Leica QWin Pro version 3.4.0, Leica Microsystems, Mannheim, Germany) in five non-overlapping high-power fields. The area fraction was calculated by dividing the positively stained surface area by the total surface area of the muscle layer. The average area fraction of the five high-power fields per animal is given as fold increase over the control value. The control value will be stated at one. All area fraction measurements were performed by one investigator blinded to the study groups.

### RNA extraction and real-time PCR

TRI reagent (Invitrogen)/chloroform extraction was used to extract RNA from snap frozen terminal ileum. Afterwards RNA was reverse transcribed into cDNA using sensifast cDNA Synthese kit (Bioline, London, UK). Quantitative real-time PCR (qPCR) was performed with the specific primers in Sensimix SYBR & Fluorescein Kit (Bioline) using a 384-wells qPCR plate. qPCR reactions were performed in a LightCycler 480 Instrument (Roche Applied Science, Basel, Switzerland) for 45 cycles. Gene expression levels of tumor necrosis factor alpha (TNF-α), IL-8 and IL-10 were determined to assess terminal ileum inflammation. mRNA expression levels of neuronal nitric oxide synthase (nNOS) and choline acetyltransferase (CHAT) were determined to assess ENS motility signaling function. LinRegPCR software (version 2016.0, Heart Failure Research Center, Academic Medical Center, Amsterdam, the Netherlands) was used for qPCR data processing. The geometric mean of the expression levels of three reference genes (ribosomal protein S15 (RPS15), glyceraldehyde 3-phosphate dehydrogenase (GAPDH) and peptidylprolyl isomerase A (PPIA)) were calculated and used as a normalization factor. Data are expressed as fold increase over the control value. Sequences of the primers used are shown in Table 1.

**Table 1:** Primer sequences

Primer	Forward	Reverse
RPS15	5′-CGAGATGGTGGGCAGCAT-3’	5′-GCTTGATTTCCACCTGGTTGA-3’
GAPDH	5′-GGAAGCTCACTGGCATGGC-3’	5′-CCTGCTTCACCACCTTCTTG-3’
PPIA	5′-TTATAAAGGTTCCTGCTTTCACAGAA-3’	5′-ATGGACTTGCCACCAGTACCA-3’
IL-8	5′-GTTCCAAGCTGGCTGTTGCT-3’	5′-GTGGAAAGGTGTGGAATGTGTTT-3’
IL-10	5′-CATGGGCCTGACATCAAGGA-3’	5′-CGGAGGGTCTTCAGCTTCTC-3’
TNF-α	5′-GCCGGAATACCTGGACTATGC-3’	5′-CAGGGCGATGATCCCAAAGTAG-3’
nNOS	5′-CGGCTTTGGGGGTTATCAGT-3’	5′-TTGCCCCATTTCCACTCCTC-3’
CHAT	5′-CCGCTGGTATGACAAGTCCC-3’	5′-GCTGGTCTTCACCATGTGCT-3’

### Data analysis

Statistical analyses were performed using GraphPad Prism (version 6.01, GraphPad Software Inc., La Jolla, CA, USA). Data are presented as median with interquartile range. Differences between the groups and the controls were analyzed using a nonparametric Kruskal–Wallis test followed by Dunn’s post hoc test. Differences are considered statistically significant at *p* ≤ 0.05. Differences with a *p* < 0.10 are also taken into account because of the small study groups and because of potential biological relevance, and described as tendencies as previously described (Willems et al. 2016). This assumption will decrease the chance of a type II error, but increases the chance of a type I error.

## Results

### Chorioamnionitis induced intestinal inflammation

A statistically significant increase in MPO-positive cells was seen in the mucosa 4 and 8 days after IA LPS exposure, compared to control (*p* < 0.05; Table 2).

**Table 2:** Immune cells count in the mucosal layer

	Control (n = 6)	5 h LPS (***n*** = 6)	12 h LPS (n = 7)	24 h LPS (***n*** = 7)	2d LPS (n = 6)	4d LPS (n = 6)	8d LPS (n = 7)	15d LPS (n = 6)
MPO+ cell count	102	74	159	76	151	354*	332*	224
SD (±)	110	77	166	51	105	**162**	**101**	96

Values are expressed as median numbers of cells per square millimeter. SD: Standard deviation. Kruskal–Wallis test with Dunn’s post hoc test was performed. * *p* < 0.05 compared to control

**Fig. 2:**
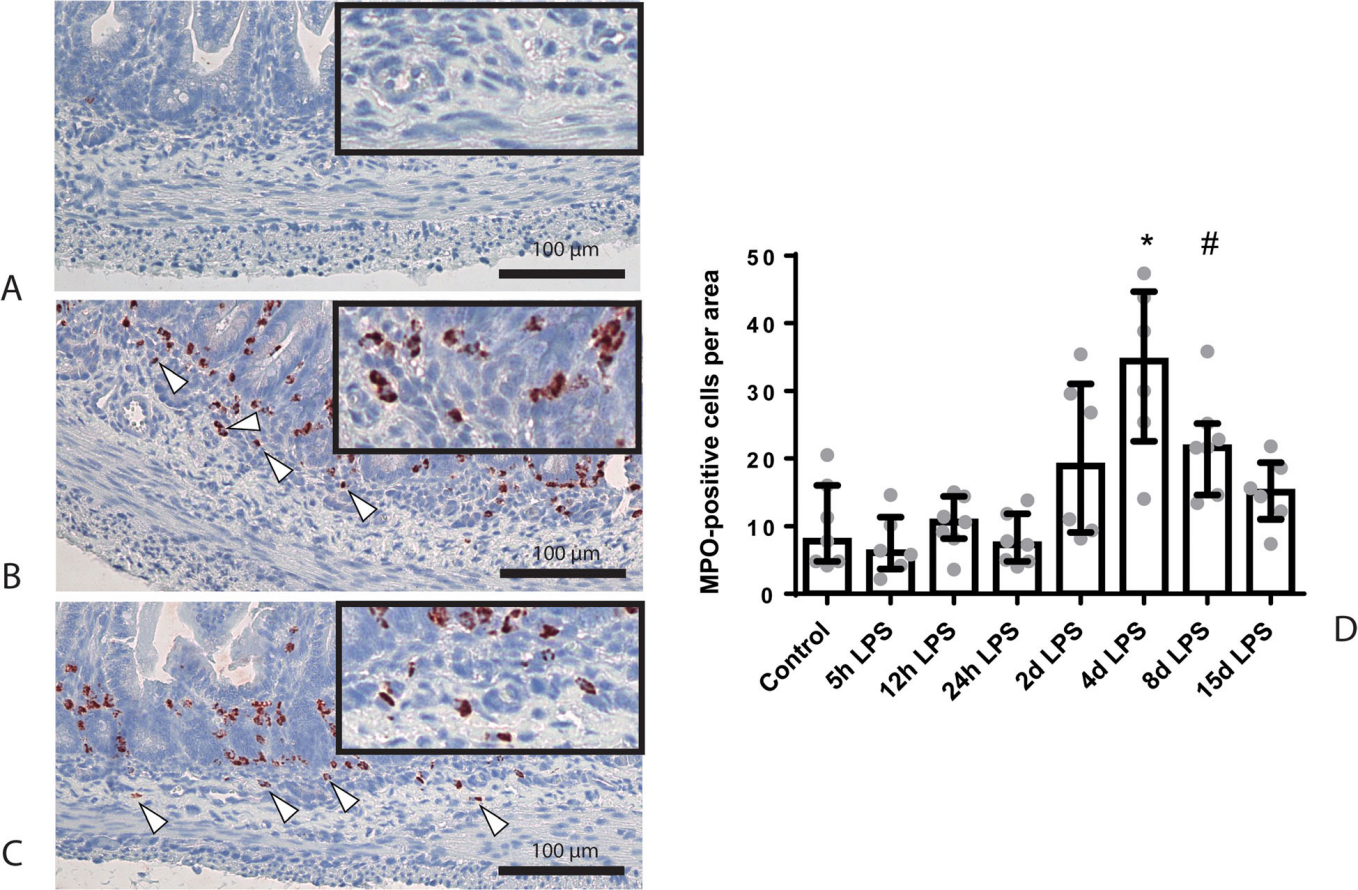
Representative images of submucosal neutrophil influx reflected by MPO-positive cell (indicated by white triangles) counts of the control (**a**), 4 days of IA LPS (**b**) and 8 days of IA LPS group (**c**). **d** Increased MPO count in animals exposed to 4 and 8 days of IA LPS. * *p* < 0.01 compared to control. # *p* = 0.08 compared to control

In the submucosa, there was an increase of MPO-positive cells in animals exposed to 4 days of IA LPS, and submucosal MPO-positive cells still tended to be increased after 8 days of IA LPS exposure, compared to control (*p* < 0.05 and *p* = 0.08; Fig. 2).

**Fig. 3:**
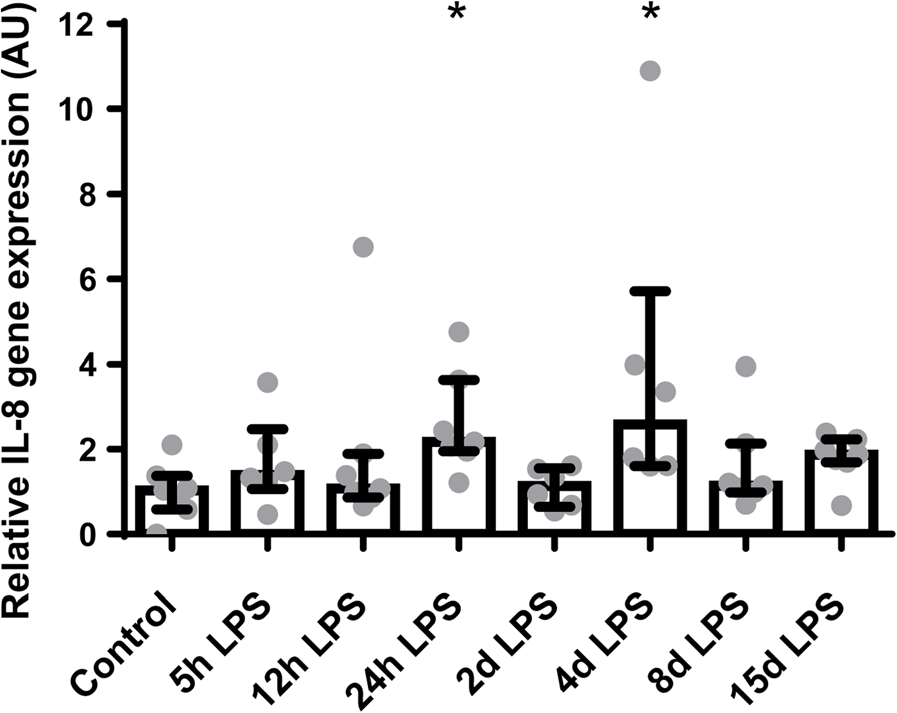
Relative gene expression of IL-8 in arbitrary unit (AU). Increased IL-8 gene expression in animals exposed to 24 h and 4 days of IA LPS. * *p* < 0.05 compared to control

Examination of underlying cytokine levels revealed increased ileal IL-8 mRNA levels after 24 h and 4 days of IA LPS exposure, compared to control (both *p* < 0.05; Fig. 3). No differences were seen in IL-10 and TNF-α mRNA levels, compared to control (Additional file 1).

### Chorioamnionitis induced enteric nervous system alterations

**Fig. 4:**
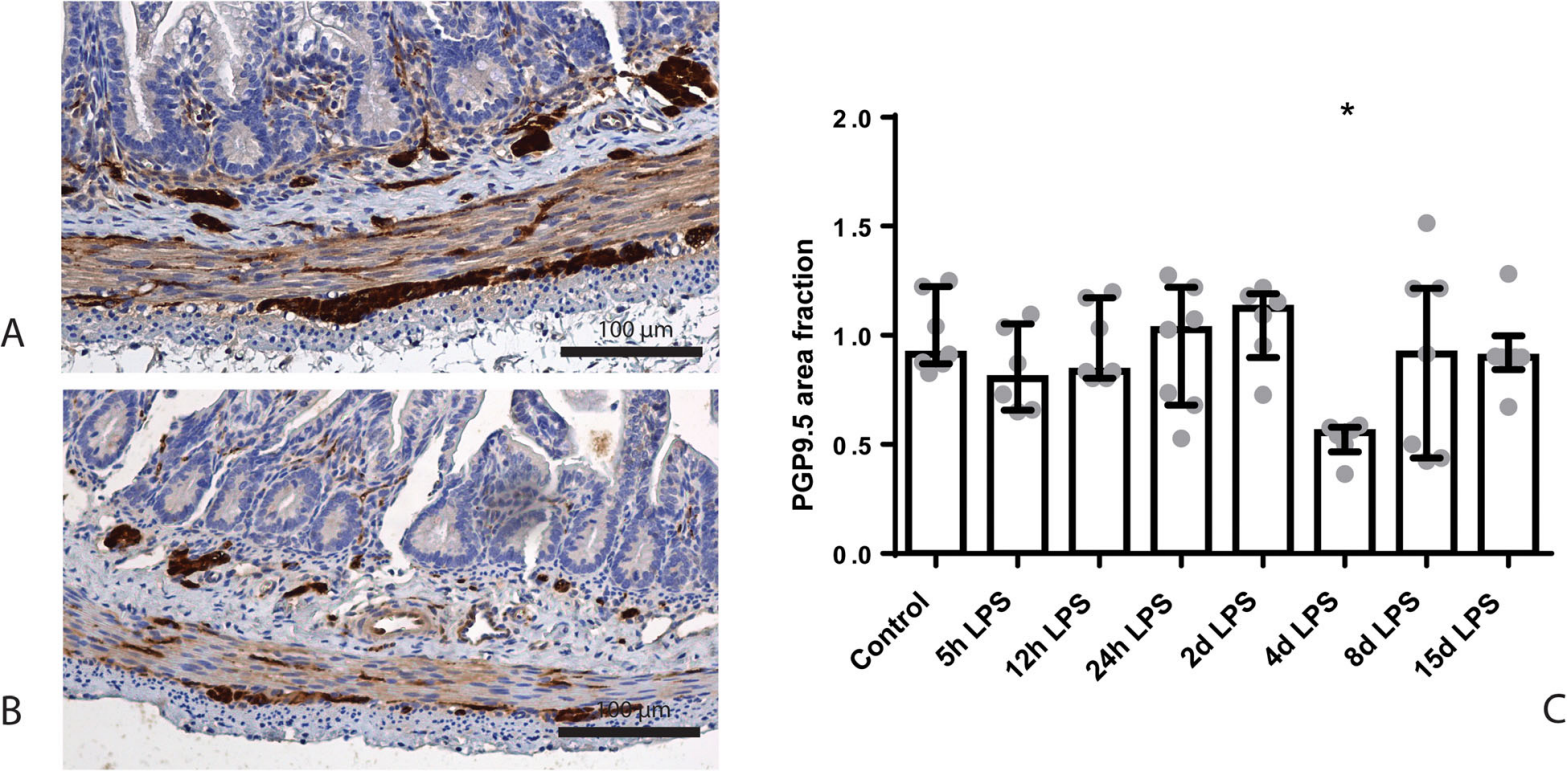
Representative images of PGP9.5 immunoreactivity in the submucosal and myenteric plexus of the control (**a**) and 4 days IA LPS group (**b**). Area fraction of PGP9.5 in the myenteric plexus (**c**) as fold increase over the control value. **c** PGP9.5-positive surface area was decreased in animals exposed to 4 days of IA LPS. * *p* < 0.05 compared to control

**Fig. 5:**
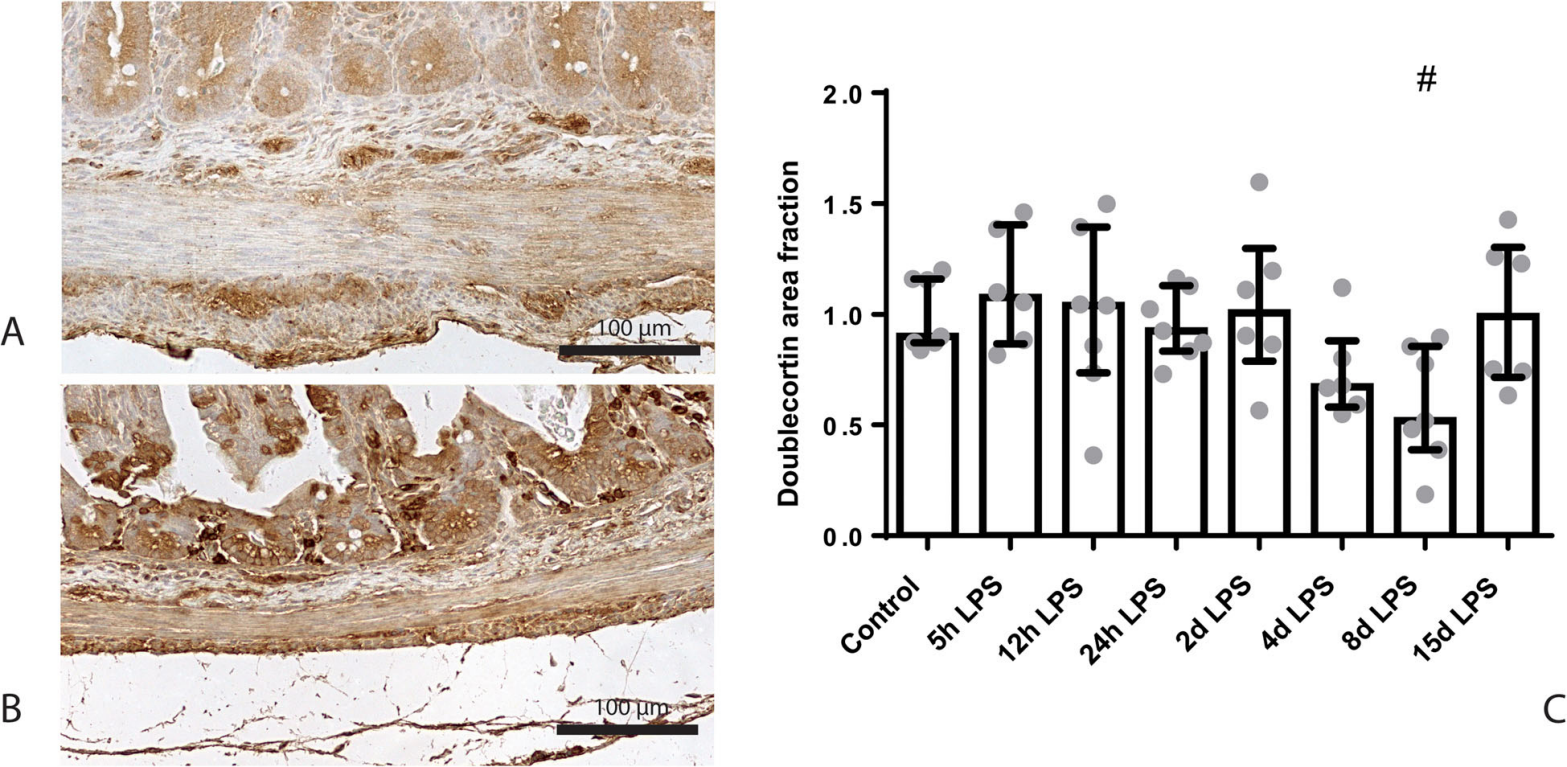
Representative images of doublecortin immunoreactivity in the submucosal and myenteric plexus of the control (**a**) and 8 days of IA LPS group (**b**). Area fraction of doublecortin in the myenteric plexus (**c**) as fold increase over the control value. **c** Doublecortin-positive surface area tended to be decreased in animals exposed to 8 days of IA LPS. # *p* = 0.07 compared to control

The PGP9.5-positive and doublecortin-positive surface areas in the submucosal plexus were unchanged in all groups compared to control (Additional file 2). In the myenteric plexus, the PGP9.5-positive surface area was decreased after 4 days of IA LPS exposure, compared to control (*p* < 0.05; Fig. 4). This reduction was resolved after 8 days of IA LPS exposure. At this time point, the doublecortin-positive surface area tended to be decreased in the myenteric plexus of LPS exposed animals, compared to control (*p* = 0.07; Fig. 5).

**Fig. 6:**
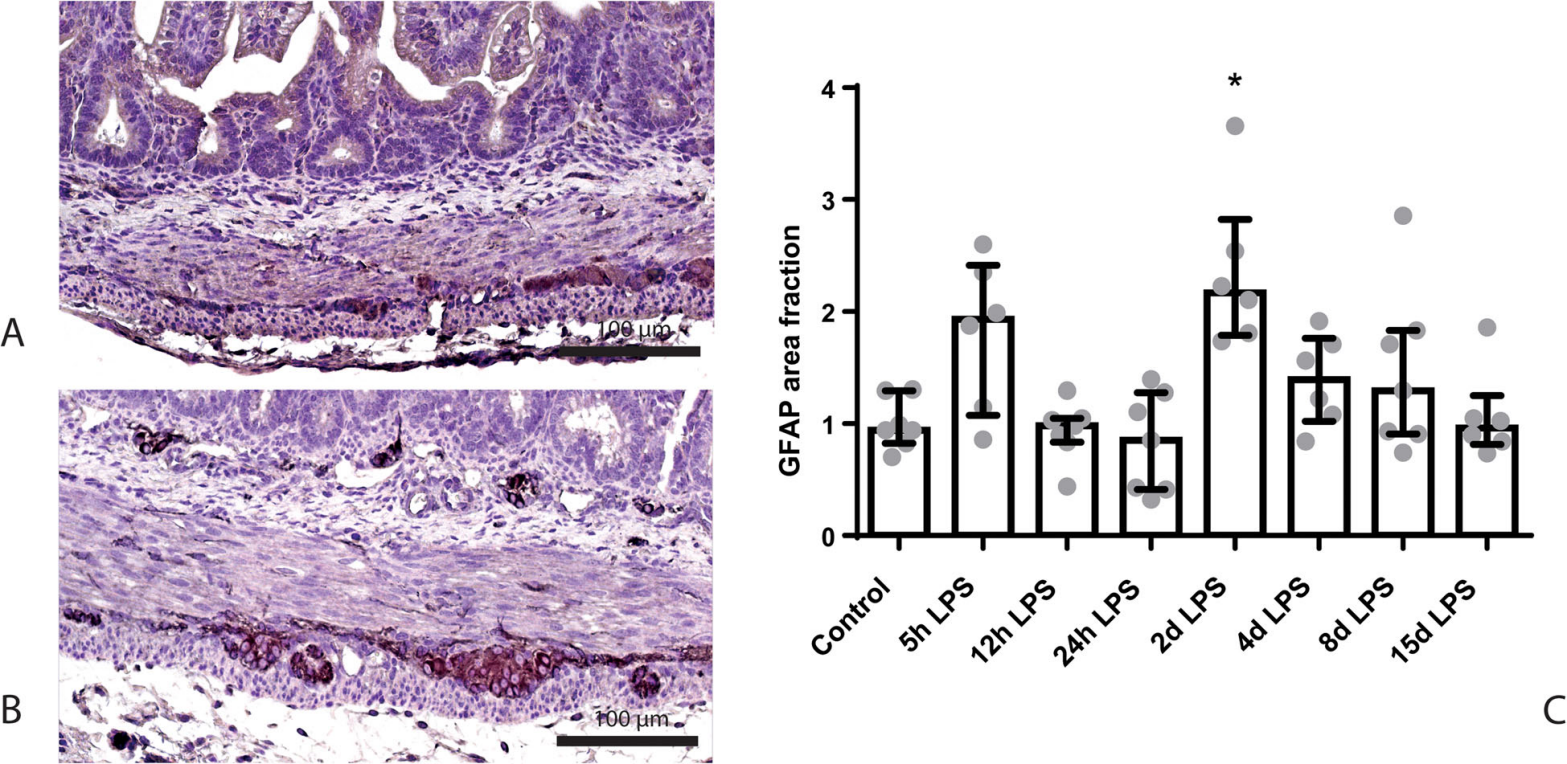
Representative images of GFAP immunoreactivity in the submucosal and myenteric plexus of the control (**a**) and 2 days of IA LPS group (**b**). Area fraction of GFAP in the myenteric plexus (**c**) as fold increase over the control value. **c** GFAP-positive surface area in the myenteric plexus was increased in animals exposed to 2 days of IA LPS. * *p* < 0.05 compared to control

In the submucosal plexus, no differences in the GFAP-positive surface areas were observed (Additional file 3), while in the myenteric plexus, the GFAP-positive surface area was increased in animals exposed to 2 days of IA LPS, compared to control (*p* < 0.05; Fig. 6).

**Fig. 7:**
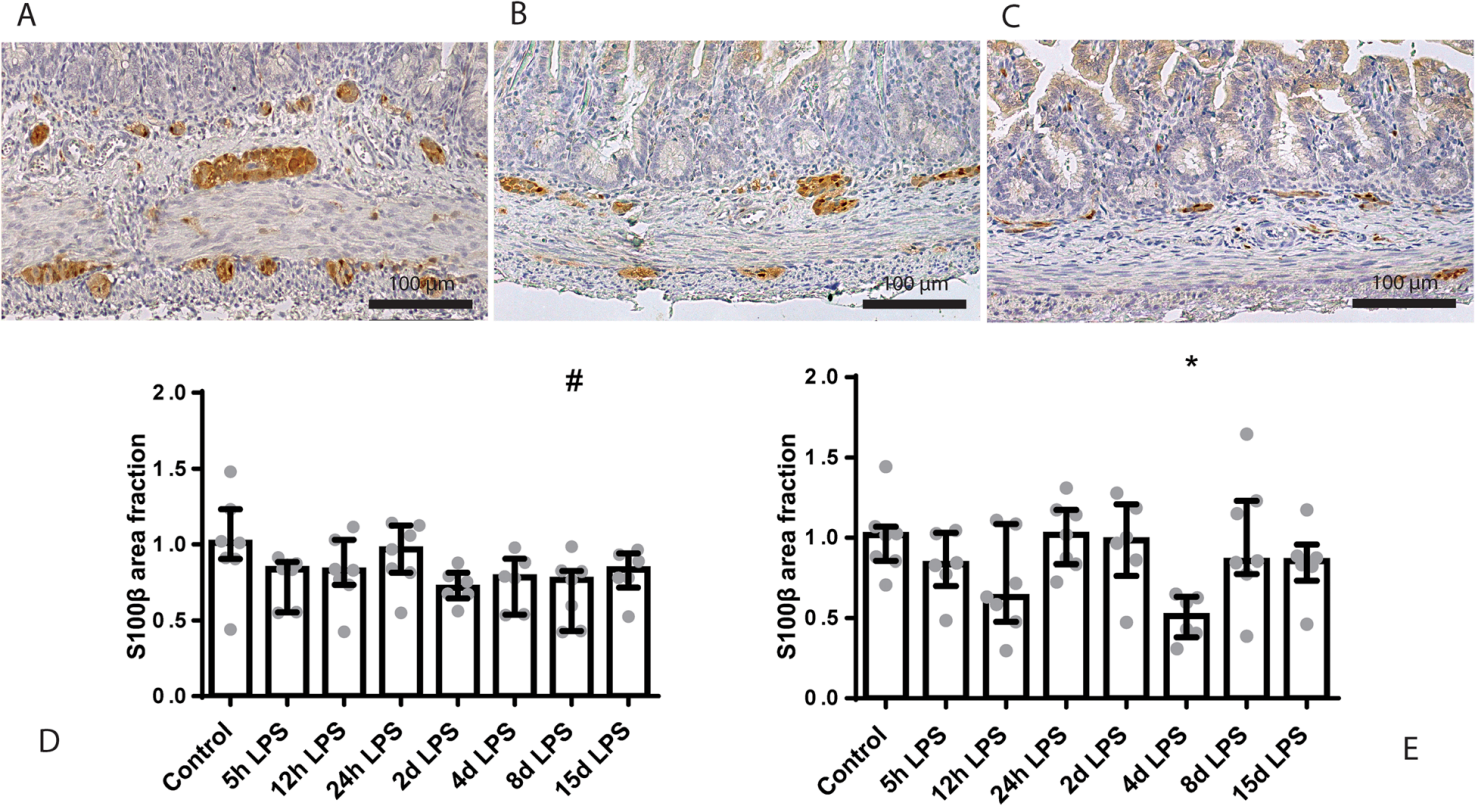
Representative images of S100β immunoreactivity in the submucosal and myenteric plexus of the control (**a**), 4 days of IA LPS (**b**) and 8 days of IA LPS group (**c**). Area fraction of S100β in the submucosal (**d**) and myenteric plexus (**e**) as fold increase over the control value. **d** S100β-positive surface area tended to be decreased in animals exposed to 8 days of IA LPS. # *p* = 0.09 compared to control. **e** S100β-positive surface area is decreased in animals exposed to 4 days of IA LPS. * *p* < 0.05 compared to control

The S100β-positive surface area in the submucosal plexus tended to be decreased in animals exposed to 8 days of IA LPS, compared to control (*p* = 0.09; Fig. 7). In the myenteric plexus, the S100β-positive surface area was decreased in animals exposed to 4 days of IA LPS, compared to control (*p* < 0.05; Fig. 7).

No differences in nNOS and CHAT mRNA expression were observed between the groups (Additional file 4).

## Discussion

In the current study, mucosal and submucosal intestinal inflammation was observed in the terminal ileum after 4 days of IA LPS exposure. On mRNA level, gut inflammation (IL-8) also occurs after 24 h of IA LPS exposure, and this time point overlaps with the fetal systemic immune response, characterized by increased circulatory IL-6 levels (Gussenhoven et al. 2018). In utero gastro-intestinal transit studies showed it takes approximately 24 h for the swallowed AF to reach the mid-ileum (unpublished findings). Hence, this early inflammatory response in the terminal ileum is probably not caused by a local process, but solely the result of fetal systemic inflammation. In line, previous research in the same ovine model has shown that chorioamnionitis induced gut inflammation is the combined effect of direct gut exposure to LPS and a lung-mediated systemic inflammatory response (Wolfs et al. 2014). It is possible that the early intestinal IL-8 peak contributes to the submucosal and mucosal increase of inflammatory cells at 4 days of IA LPS exposure through stimulation of chemotaxis (Russo et al. 2014).

Interestingly, the most evident signs of ENS alterations were also seen after 4 days, and after 8 days of IA LPS exposure. After 4 days IA LPS exposure, the myenteric plexus PGP9.5-positive surface area was decreased, indicating a loss of enteric neurons and/or reduction of PGP9.5 immunoreactivity of enteric nerve cells. Since the doublecortin-positive (immature neurons) surface area remained unchanged, this was probably the result of affected mature neurons. The reduced PGP9.5-positive surface area after 4 days of IA LPS exposure was recovered after 8 days of IA LPS exposure. The doublecortin-positive surface area tended to decrease at this time point. These findings might indicate that an initial loss of mature neurons is compensated by an accelerated maturation of immature neurons. Whether such an accelerated maturation is sufficient to fully compensate for the identified loss of neurons remains to be elucidated. These findings combined with the unaltered PGP9.5-positive and doublecortin-positive surface area after 2 and 7 days of IA LPS exposure in a previous study (Heymans et al. 2020), show that the ENS changes found are time-dependent and may recover following prolonged intrauterine inflammation. Interestingly, in a previous study, a similar loss of mature neurons was observed after chronic IA exposure to UP, indicating that different inflammatory triggers can induce similar ENS damage (Heymans et al. 2020).

Enteric glial cells are important for neuronal maintenance, survival, and function (De Giorgio et al. 2012), and are capable of generating enteric neurons in response to injury (Joseph et al. 2011; Laranjeira et al. 2011). In addition, enteric glia respond, in a manner similar as reactive astrogliosis in the central nervous system, to ENS injury and inflammation by changing both their morphology and their expression of key proteins such as GFAP (Boesmans et al. 2015; Rosenbaum et al. 2016). The neuronal loss in the myenteric plexus after 4 days of LPS exposure is accompanied with a reduced S100β-positive surface area, likely representing a loss of glial cells and/or loss of S100β immunoreactivity within glial cells, as was earlier described during chronic IA UP exposure (Heymans et al. 2020). Interestingly, this loss of neurons and glial cells is preceded by an increased myenteric plexus GFAP immunoreactivity after 2 days of LPS exposure. It is likely that the observed glial cell response results from fetal systemic inflammation and/or intestinal inflammation, since pro-inflammatory cytokines have been shown to induce GFAP expression in enteric glial cells (von Boyen et al. 2004). Moreover, as activated enteric glial cells can secrete various cytokines and other mediators involved in the infiltration and activation of immune cells (Stoffels et al. 2014; Sharkey 2015), the observed glial cell reaction can contribute to the intestinal influx of neutrophils observed after 4 days of IA LPS exposure. Since a glial cell response in the context of intestinal inflammation can be destructive (Brown et al. 2016) and eventually neuroregenerative (Belkind-Gerson et al. 2017), it is to date unclear whether it contributes to the loss of neurons and glial cells, or is a protective mechanism that falls short with prolonged inflammation.

In this study, the most profound ENS changes were found in the myenteric plexus, rather than the submucosal plexus. This is in concordance with earlier findings in fetal lambs that were chronically IA exposed to UP (Heymans et al. 2020). Moreover, inflammation driven pathological changes of the ENS are more often found in the myenteric plexus than in the submucosal plexus (De Giorgio et al. 2004). The mechanisms behind this apparent increased vulnerability of the myenteric plexus remain to be elucidated. At present, we can only speculate about the mechanisms responsible for the observed differences because multiple possible explanations are in play. First, since the ENS undergoes rapid structural growth in utero, the composition of the submucosal and myenteric plexus might be differently altered by the combination of ongoing developmental processes and LPS exposure. Alternatively, the migratory pattern of cells in these plexi might be different during this essential developmental period of the ENS. Second, the macrophages in the plexus, which are in close proximity to neuronal cell bodies and nerve fibers, undergo differentiation towards a multitude of subsets depending on microenvironment but also depending on developmental stage and bacterial colonization. Our findings indicate that these cells play a role in the differential response of the submucosal and myenteric plexus, although the reason for that remains speculative. Notably, the transcriptional profiles of macrophages gradually differ from the lumen to the myenteric plexus. As a result, the macrophages closer to the lumen play an important role by sampling luminal bacteria and initiating adaptive immune responses to clear pathogenic bacteria, whereas macrophages in the muscularis, which are comparatively more distant from luminal stimulation, are primarily involved in tissue protection and regulation of the activity of enteric neurons and peristalsis (Gabanyi et al. 2016; De Schepper et al. 2018). It is tempting to speculate that phenotypical differences of these immune cells following exposure to a bacterial stimulus in the different plexi are involved in the observed differences between the submucosal and myenteric plexus.

At present, it is unclear whether the observed changes have postnatal functional consequences. As the mRNA expression of CHAT and nNOS are unchanged, in utero motility signaling function could be unaltered. This confirms and extends previous findings in fetal lambs chronically IA exposed to UP (Heymans et al. 2020). The resolved inflammation and the recovery of (immature) neurons and glial cells after fifteen days of IA LPS exposure indicate that damage due to IA LPS exposure probably can be repaired in utero. Nevertheless, it is likely that a child that is born prematurely with ongoing inflammation due to FIRS will experience additional postnatal inflammatory stimuli such as mechanical ventilation (Bose et al. 2013) or sepsis (Machado et al. 2014). The effects of these postnatal exposures on the ENS should be studied in order to shed light on the long term consequences of (intra-uterine) inflammation for ENS development and function. Notably, 4 to 8 days after the start of intrauterine infection could very well be the window of vulnerability in which additional inflammation may have a higher impact as the ENS is already affected at this time point.

A limitation of this study is the relatively low number of animals per group, which is an unavoidable shortcoming of the translational large animal model. Secondly, the current set-up with the fixed moment of premature birth does not exclude a potential influence of gestational age at start of intrauterine infection. Thirdly, in the current study we were unable to unravel the mechanisms behind the observed changes, as no serial sampling was applied following a specific injection time point.

## Conclusions

In the current study, submucosal intestinal inflammation was detected after 4 days of IA LPS exposure that coincided with gut mucosal and fetal systemic inflammation. At the same time point, a loss of PGP9.5 and S100β immunoreactivity in the myenteric plexus was observed. These changes are preceded by a glial cell response with systemic inflammation and local gut inflammation. Although initial ENS damage seemed to recover after prolonged IA LPS exposure, additional postnatal inflammatory hits that a premature born child is likely to encounter may further harm the ENS and influence functional outcomes. In this context, 4 to 8 days after the start of intrauterine inflammation may be a window of increased ENS vulnerability, indicating that therapeutic interventions should ideally start before or at this time point.

## Supplementary information

**Additional file 1.** Relative gene expression of IL-10 and TNF-α in arbitrary unit (AU). No differences were seen in IL-10 and TNF-α mRNA levels, compared to control.

**Additional file 2.** Area fraction of PGP9.5 (A) and doublecortin (B) in the submucosal plexus (C) as fold increase over the control value. The PGP9.5-positive and doublecortin-positive surface areas in the submucosal plexus were unchanged in all groups compared to control.

**Additional file 3.** Area fraction of GFAP in the submucosal plexus as fold increase over the control value. No differences in the GFAP-positive surface areas were observed in the submucosal plexus compared to control.

**Additional file 4.** Relative gene expression of nNOS and CHAT in arbitrary unit (AU). No differences in nNOS and CHAT mRNA expression were observed between the groups.

## Data Availability

The datasets used and/or analysed during the current study are available from the corresponding author on reasonable request.

## Abbreviations

AF: Amniotic fluid
AU: Arbitrary unit
BSA: Bovine serum albumin
CHAT: Choline acetyltransferase
ENS: Enteric nervous system
FCS: Fetal calf serum
FIRS: Fetal inflammatory response syndrome
GAPDH: Glyceraldehyde 3-phosphate dehydrogenase
GFAP: Glial fibrillary acidic protein
IA: Intra-amniotic
IL: Interleukin
LPS: Lipopolysaccharide
MPO: Myeloperoxidase
NEC: Necrotizing enterocolitis
NGS: Normal goat serum
nNOS: Neuronal nitric oxide synthase
PBS: Phosphorylated buffer saline
PGP9.5: Protein gene product 9.5
PPIA: Peptidylprolyl isomerase A
RPS15: Ribosomal protein S15
TNF-α: Tumor necrosis factor alpha
