# Supplementary figures and images for: Chorioamnionitis induces enteric nervous system injury: effects of timing and inflammation in the ovine fetus
Source: Mol Med. 2020 Sep 3;26:82. doi: 10.1186/s10020-020-00206-x (PMC7469100; doi:10.1186/s10020-020-00206-x)

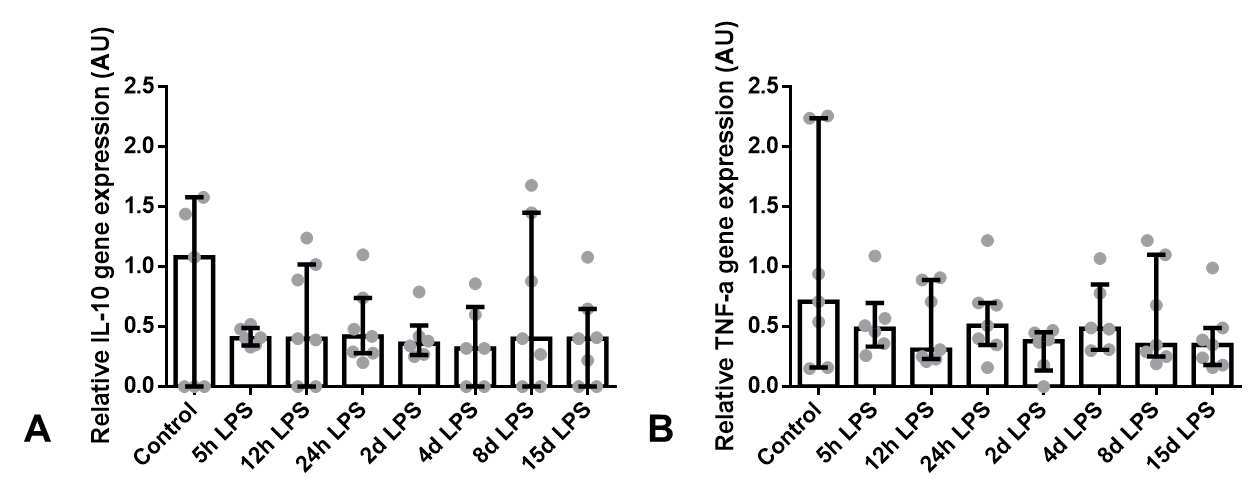

Supplement: Supplementary file 1 — Additional file 1. Relative gene expression of IL-10 and TNF-α in arbitrary unit (AU). No differences were seen in IL-10 and TNF-α mRNA levels, compared to control. [file 10020_2020_206_MOESM1_ESM.tiff]

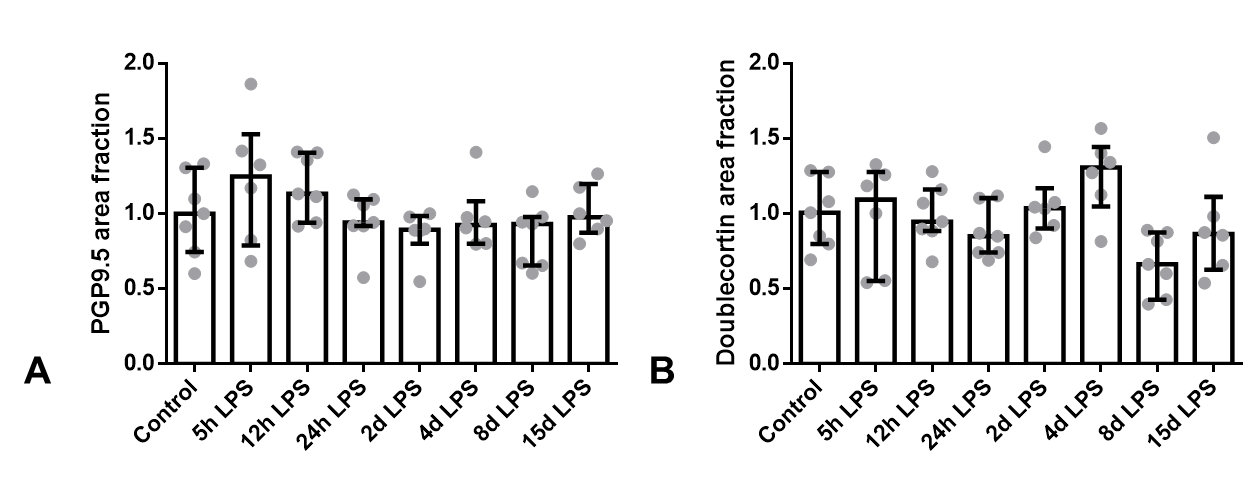

Supplement: Supplementary file 2 — Additional file 2. Area fraction of PGP9.5 (A) and doublecortin (B) in the submucosal plexus (C) as fold increase over the control value. The PGP9.5-positive and doublecortin-positive surface areas in the submucosal plexus were unchanged in all groups compared to control. [file 10020_2020_206_MOESM2_ESM.tiff]

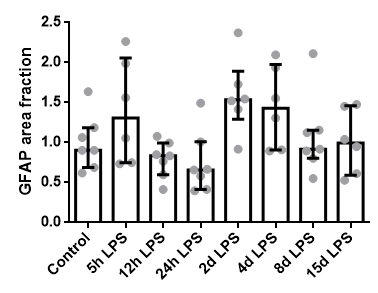

Supplement: Supplementary file 3 — Additional file 3. Area fraction of GFAP in the submucosal plexus as fold increase over the control value. No differences in the GFAP-positive surface areas were observed in the submucosal plexus compared to control. [file 10020_2020_206_MOESM3_ESM.tiff]

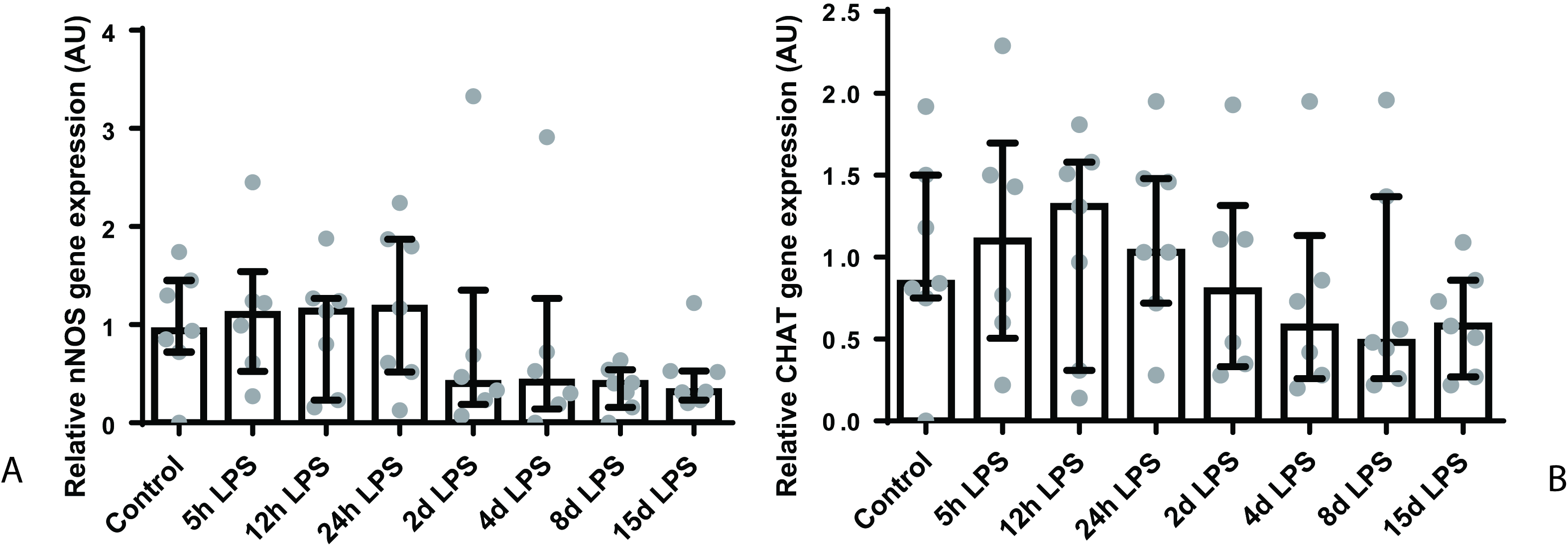

Supplement: Supplementary file 4 — Additional file 4. Relative gene expression of nNOS and CHAT in arbitrary unit (AU). No differences in nNOS and CHAT mRNA expression were observed between the groups. [file 10020_2020_206_MOESM4_ESM.tif]
